# Suppressing the coffee-ring effect of colloidal droplets by dispersed cellulose nanofibers

**DOI:** 10.1080/14686996.2017.1314776

**Published:** 2017-05-09

**Authors:** Yuto Ooi, Itsuo Hanasaki, Daiki Mizumura, Yu Matsuda

**Affiliations:** ^a^ Institute of Engineering, Tokyo University of Agriculture and Technology, Tokyo, Japan.; ^b^ Institute of Materials and Systems for Sustainability, Nagoya University, Nagoya, Japan.

**Keywords:** Coffee ring, colloid, particle, cellulose, nanofiber, ink, drop, film, soft matter, mesoscale

## Abstract

We report that the addition of a small amount of cellulose nanofibers (CNFs) into an aqueous dispersion of colloidal particles suppresses the coffee-ring effect when the dispersion dries on a solid substrate, as revealed by the computational analysis of experimental time-series images and by particle image velocimetry. The addition of CNFs is much more effective than the increase of colloidal particle concentration at the same weight percentage; it is also more environment friendly than the use of typical molecular surfactants. This finding is promising for the fabrication of metamaterials from colloidal dispersions and for ink printing in electronics, where CNFs can also serve as a substrate for flexible devices.

## Graphical Abstract

**Figure:**
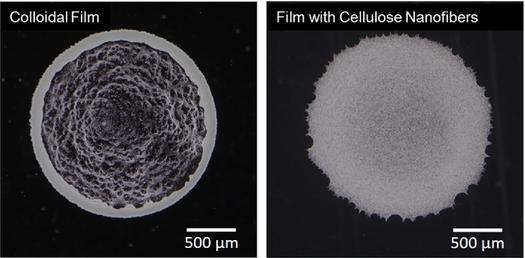

## 1. Introduction

Many industrial products involve drying of suspensions of small particles. Pigment inks are typical examples, where particles with specified colors are dispersed in liquid. The drying of the dispersant liquid of the inks discharged from the nozzles of inkjet printers leaves thin films of accumulated particles on a solid surface, such as paper. The application of inkjet printing is not limited to drawing characters and pictures or protection of bare solid surfaces but includes the drawing of electric circuits in printed electronics [1–4]. The drying of particle dispersion is not limited to ink and printing. One of the related intensive research areas is the fabrication of colloidal photonic crystal, which is the assembled structure of colloidal particles typically in regular lattice patterns. They may find applications in pH sensors [5] and laser sources with variable wavelength [6]. Regardless of top-down or bottom-up approaches, photonic crystals with specific optical properties are designed by the unit of structure, unlike atoms or molecules. The patterning of particles larger than atoms or molecules are now one of the popular techniques to fabricate new materials in general, and they are often referred to as ‘metamaterials’, where the self-assembly of particles from drying suspension often plays an important role [7–10]. Thus, drying of particle dispersion is widely exploited in scientific research and industry.

However, there are some typical problems that engineers and scientists encounter in the wide use of drying phenomena of colloidal particles. One of the most well-known issues is the so-called coffee-ring effect [11–16]. Its name originates from the effect that can be observed after drying of a colloidal dispersion, such as coffee. Drying of a droplet with particles often exhibits ‘pinning’ [17,18], where particles stays on the periphery of the gas–liquid–solid interface. The suspended particles tend to be transported to the periphery through the drying process. After the dispersant dries up, the particles form a ring-shaped aggregation. The point is not the circular ring but the non-uniform distribution of particles, where the inner part of the droplet endsup at a lower density. The non-uniform thickness of the colloidal film degrades the coating of paints and clarity of printed characters on paper, and the shapes of cross sections of particulate films substantially affect the electric conductivity in printed electronics [19,20]. The conducting wires are desired to have as low electric resistance as possible, and the elements of a heater should have a specified value of resistance [4]. Thus, the problem of uniformity in colloidal film fabrication is not only a matter of appearance but also a serious issue of functionality in the wide industrial context.

The conventional methodology of suppressing the coffee ring effect is based on the use of various kinds of organic compounds in order to tune the viscosity, surface tension, the speed of drying of dispersant, and the Debye length of the particles. There are diverse recipes for numerous applications, but environmental friendliness has been an important outstanding issue [21,22]. Recently, it has been reported that controlling the shape of particles, more specifically changing spherical particles into prolate ones, can prevent the coffee-ring effect [23]. The non-spherical shape of the particles leads to the characteristic capillary effect and anisotropy in Brownian motion [24,25]. While the control of shape is promising when it is possible, it is not trivial to apply the technique to arbitrary materials. Nevertheless, there is a possibility to suppress the coffee-ring effect by physical means at mesoscopic space scale between the molecules and full continuum in mechanical terms. While particle distribution with negligible effect of transient flow is often dominated by the free-energy of interactions between the particles [26], the coexistence of flow and Brownian motion can affect the patterns of molecular network on a substrate [27], and this is the essence of the coffee-ring effect [14]. The mesoscopic characteristics of materials can affect this balance. In this article, we propose an alternative technique to suppress the coffee-ring effect in the formation of colloidal film formation by drying up of particle dispersion. We employ cellulose nanofibers (CNFs) as a substitute for molecular additives.

Chemical constituents of CNFs are basically the same as the main ingredient of ordinary paper, i.e. a kind of biomass usually made from wooden pulp [28]. CNF is an environmentally friendly and abundant material. CNFs differ from raw materials of ordinary paper by their much smaller nominal diameters of tens of nanometers. Consequently CNF can be processed into transparent paper [29,30]. Their mechanical strength is also attracting attention, and there are some reports on the improvements of mechanical property through the addition of CNFs into resin and rubber [31]. If the use of CNFs for the above-mentioned purpose is effective, the fabricated films might acquire this advantageous mechanical property as well. The addition of CNFs may alter the electrical resistivity of conductive wires in printed electronics, but the fine tuning of the concentration might be exploited for the control of electric resistivity itself. Sufficiently small diameters of CNFs may be compatible with the application for photonic materials. In this article, we show that the use of CNFs indeed suppresses the coffee-ring effect, and it has significant effect compared to the mere tuning of particle concentration. We quantify the suppression of the coffee-ring effect by CNF through computational analysis of time-series image data obtained from experimental measurements. Clear understanding of the phenomena in the fabrication processes will be useful in diverse specific applications where mechanical properties and electrical conductivity are the main concerns.

**Figure 1.**
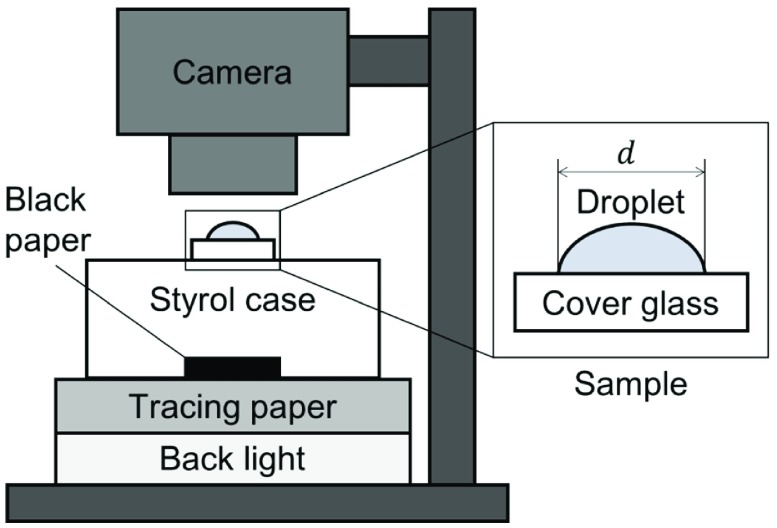
Schematic diagram of the experimental setup for time-lapse measurement by digital camera. The sample of a droplet on a cover glass was placed on a transparent styrol case where black paper was located beneath the sample. The brightness of the LED backlight was mediated by tracing paper.

**Table 1.** Concentrations 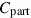 and 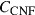 of polystyrene particles and CNFs in water for each sample, respectively. The environmental conditions and drying durations 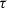 are also summarized. Time and length were normalized in the analysis by 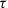 and *d*, respectively. The measurement error of the initial droplet diameter *d* (cf. Figure 1) on the substrate was ca. 0.007 mm. RH stands for relative humidity.

Sample label	(wt%)	(wt%)	*d* (mm)	Temp. (C)	RH (%)	(s)
A	0.1	0	1.960	18.6	60	1331
B	0.1	0.01	1.940	16.7	42	881
C	0.1	0.1	1.960	19.2	59	1380
D	0.1	1.0	1.910	18.6	60	1201
E	0.2	0	1.910	16.7	42	940

## 2. Experimental methods

We study the samples of colloidal particle suspension with and without CNFs. We employ a commercially available CNF (FMa-10002, BiNFi-s, Sugino Machine Limited, Toyama, Japan) [32], which is an aqueous dispersion of CNF fabricated through mechanical processing of raw material. The typical fiber diameter and length are ca. 20 nm (more than 90% of the fibers) and 1 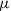
m, respectively, as revealed by scanning electron microscopy (SEM). We have used water for molecular biology (H20MB0106, Millipore Corp., Darmstadt, Germany) for the preparation of particle suspension. The particles are made of polystyrene with a diameter of ca. 1.4 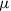
m and relative density of ca. 1.05 with respect to water (SX-130H, Chemisnow, Soken Chemical & Engineering Co., Ltd, Tokyo, Japan). Although the standard viewpoint of colloidal particle dispersion for experimental specification is based on the zeta potential and pH, we do not use these criteria in this article because the purpose of this study is suppression of the coffee-ring effect without fine-tuning of the Debye length and pH by molecular additive. The quantitativedetails can vary depending on these parameters, but one can try finite range of them after all. Therefore, we try to make the system as simple as possible in terms of sample preparation. The particles were not processed with chemical surface modification in the fabrication process. We show the distinctive effect of CNFs with these primitive chemical specifications. The concentrations of the particles and CNFs are varied as summarized in Table 1. We prepared the samples starting from the measurement of particle powders by an electronic mass scale, then dispersed in pure water. Originally 2.1 wt% of aqueous dispersion of CNF was measured by micropipette. The sample before dropping was ultrasonicated (UT-106H, Sharp Corporation, Osaka, Japan) for 20 min after shaking the container by hand. Sample dispersion of 1 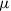
l was dropped on a cover glass (NEO cover glass, Matsunami Glass Ind., Ltd, Osaka, Japan) using a micropipette.

**Figure 2.**
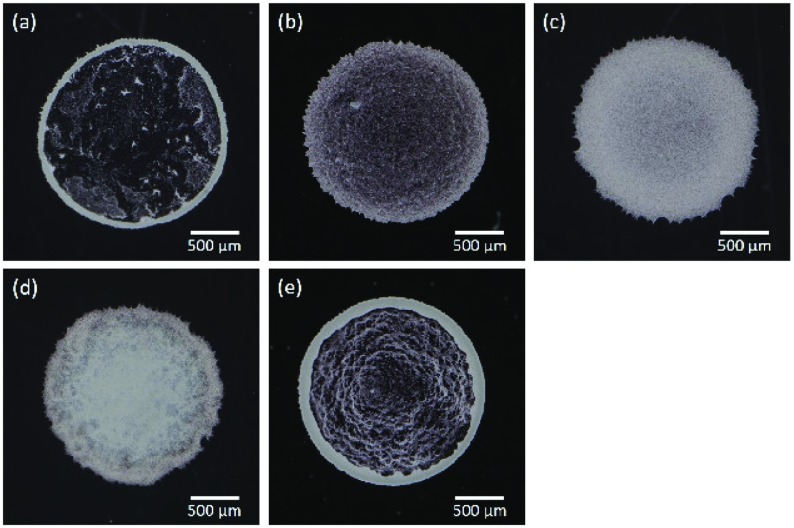
Digital camera images of dried colloidal films, where (a) to (e) corresponds to the samples A to E in Table 1, respectively. White part in each image indicates either colloidal particles or aggregated CNFs or both.

The drying droplets were observed by a camera system schematically shown in Figure 1. The droplet was observed from the top using a digital camera (DMC-GX8, LUMIX, Panasonic Corp., Osaka, Japan) in combination with a magnifying lens (NANOHA, Yasuhara Co., Ltd, Tokyo, Japan). The resolution was 1.32 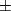 0.01 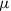
m/px based on the observation of a ruler. The particles were observed at a size in the order of 5 px. The frame rate of the camera acquisition was 1 fps and 1400 snapshots were captured. The backlight was provided by a light-emitting diode (4X5 56920, LED viewer pro, Fujifilm Corp., Tokyo, Japan), and a black background was prepared beneath the sample cover glass. The measurement was conducted in a dark room, which was air-conditioned at 20
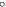
C. The possible flow of dispersed media was evaluated by particle image velocimetry (PIV) based on the normalized correlation coefficient algorithm [33]. The spatial resolution of displacements for PIV is usually in the sub-pixel order [34,35]. After we had confirmed the basic reproducibility of the overall phenomena on the qualitative level, we employed a typical sample for each condition for the time-series analysis. The length and time scale is normalized by the initial droplet diameter and the time duration till drying up (cf. Table 1). The normalization of time by 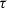 implies that we discuss the time evolution based on the stages beginning from the placing the droplet until drying up. The definition of the time point for the drying up is based on the time evolution of the time-differential images, as we will address in the next section. The droplet diameters are similar among the different samples for the discussion in this article, but this is the length parameter that affects the flow speed of the coffee-ring effect [14], and also obviously 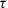.

## 3. Results and discussion

### 3.1. Film patterns

**Figure 3.**
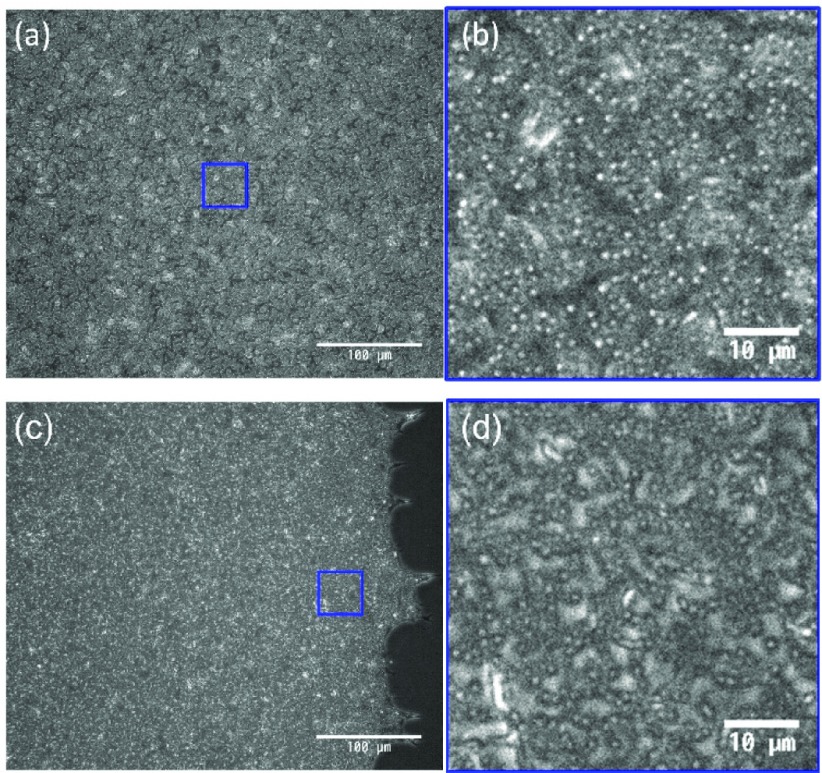
The inverted optical microscopy images of sample C. (a) and (b) are captured at the central region, and (c) and (d) are captured at the periphery of the sample, respectively. (b) and (d) are taken from the (blue) square in (a) and (c), respectively.

The digital camera images of the thin films after drying up are shown in Figure 2. Suspended particles of sample A aggregated on the periphery of the droplet and clear scarcity can be recognized inside the circular domain (Figure 2(a)). This is the consequence of the coffee-ring effect [14]. The same feature is also observed in sample E, where the particle concentration is twice as large as sample A (Figure 2(e)). On the other hand, a slight addition of CNF into the sample substantially changes the film property (Figure 2(b)). The coffee-ring effect was suppressed by the further addition of CNF as shown in Figure 2(c) and 2(d). Visual inspection of the camera images suggests that 0.1 wt% of CNF is similar to the case of 1.0 wt% except for a slightly darker image in the center compared to the periphery in sample C and non-uniformity of the pattern in the vicinity of the periphery for sample D. The typical diameter of CNF is generally smaller than the wavelength of visible light and hence invisible by ordinary optical microscopy. However, it is not certain whether it also applies to the dried film structure. Therefore, we have examined whether the apparently uniform film pattern of sample C consists of sufficient concentration of the particles at the central part of the film in comparison to that in the periphery. Figure 3 shows the inverted optical microscopy images of sample C captured at the center (Figure 3(a) and (b)) and periphery (Figure 3(c) and (d)). Although the texture looks slightly different, it is not trivial to point out the difference of particle concentrations between the center and periphery.

**Figure 4.**
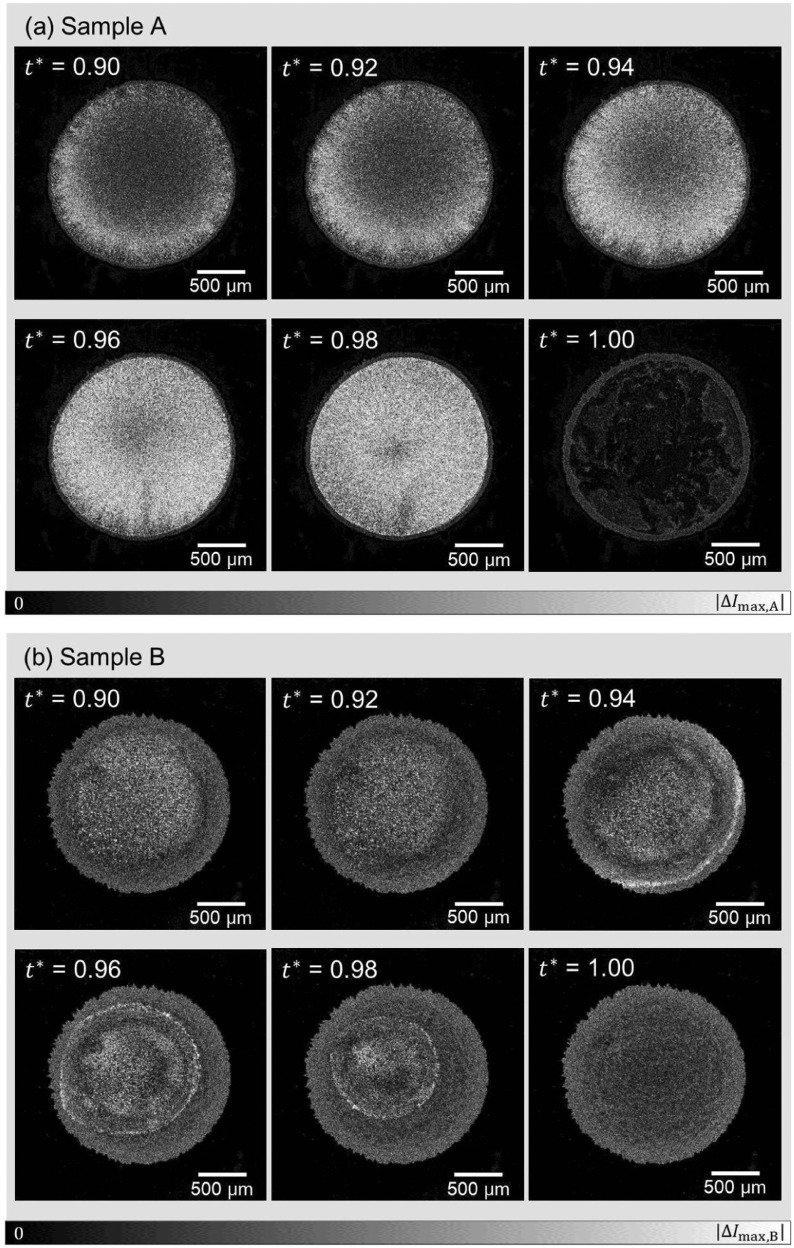
Time-differential images of samples A and B shown as a function of time. The coloring is based on the absolute value 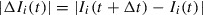, of the light intensity *I* for each pixel *i* at time *t* with a single time step difference 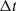, with the range from 0 to the maximum value of the pixel in the six images for each sample. The larger values of 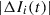 indicates the significant change of state for the respective same locations in the samples due to flow or/and evaporation.

**Figure 5.**
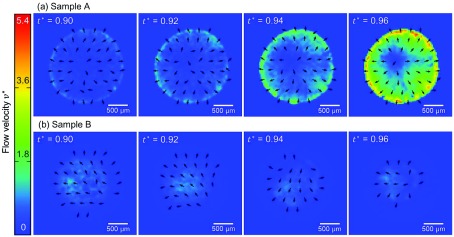
Time series images of samples A and B with visualized arrows of flow velocity fields by the PIV analysis. The arrows indicate only the direction of the flow velocity beyond the error magnitude, whereas the color mapping indicates the flow speed.

This small variation of the droplet diameter as shown in Table 1 suggests the small variation of the contact angle. Therefore, a substantial difference in dried film patterns by the addition of CNF in the colloidal dispersion is not caused by variation of surface tension. Instead, the effect of CNF is mechanical inside the droplet, at a larger scale than the molecular scale. We have confirmed that the addition of CNF is effective in the suppression of the coffee-ring effect. Differences of film property observed in the samples B, C and D compared to A does not fully answer the question of whether it is essential to use CNF - i.e. an increase in the concentration of dispersed material might also be effective. However, comparison of samples A and E shows that the increase of particle concentration from 0.1 to 0.2 does not produce a drastic difference. Furthermore, comparison between the samples C and E clarifies the effect of CNF compared to the particles themselves. Both samples C and E have the same 0.2 wt% of dispersed materials in total. Therefore, it can be concluded that the use of CNF has a remarkable effect in the suppression of the coffee-ring effect rather than just increasing the particle concentration.

### 3.2. Drying process

Now we move on to the discussion of the transient dynamic process that the films are formed by drying. Although definition of the starting point of the time evolution of drying is obvious (the moment of placing the sample droplet on the substrate by micropipette), definition of a time point of complete drying up is less clear. We solved this problem by the examination of the time-differential images as a function of time. Namely, we computed the difference of light intensity between the same location for the two images at the adjacent time steps, and monitored the time evolution of 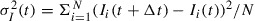, where 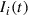 denotes the light intensity value of the image for the pixel *i* at time *t*, 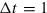 s is the frame interval of the camera, and *N* is the number of pixels in a sample image. The magnitude of 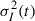 sharply drops when the drying process gets effectively completed. The consequent values of time duration 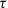 to complete the drying process for each sample were summarized in Table 1. Hereafter, we use the dimensionless time 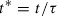 in combination with dimensionless length scaled by the droplet diameter *d*, which yields dimensionless velocity 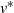.

**Figure 6.**
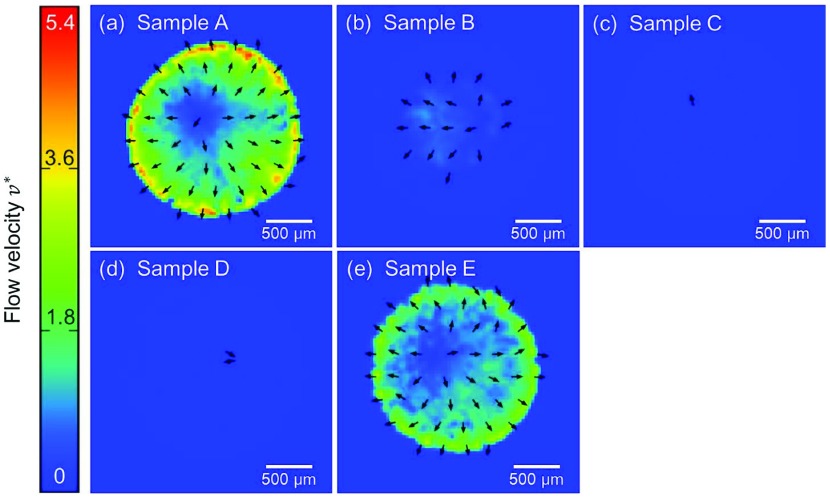
Snapshots of velocity fields based on the PIV analysis visualized by the colored arrows at dimensionless time 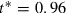 from the dropping of samples on the cover glasses. (a) to (e) corresponds to the samples A to E in Table 1, respectively.

The time evolutions of the time-differential images for samples A and B are shown in Figure 4. Sample A, which exhibited the coffee-ring phenomenon, shows significant magnitudes of time-difference values for the light intensity during the drying-up process (i.e. 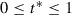). This is caused by the flow of particle dispersion and/or the time evolution of the texture for each fixed location by water evaporation. The time-differential signal is observed throughout the sample (cf. Figure 2 for the sample domain), and it almost disappears after drying up. Sample B shows qualitatively different time evolution. Substantial magnitude of time-differential signal is observed sharply at the periphery at 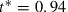, then the domain inside the closed loop of sharp signal decreases. The domain boundary is somewhat skewed from the circular shape of the initial droplet, and its center also deviates from that of the entire sample domain. The domain outside the closed loop of the sharp time-differential signal shows similar magnitude of signal to the dry state at 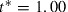. This contrasts with the clear difference in the the magnitude of time-differential signal between 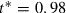 and 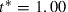 for sample A. Thus, the drying of sample B proceeds with the shrinkage of the wet subdomain inside the dry subdomain, whereas the sample A keeps the similarly wet state almost all over the sample domain until the very final stage of the drying process. It should be noted that the de-pinning and the receding contact line in the context of the coffee-ring effect is based on the material as a whole instead of the dispersant liquid component of the dispersion. The drying process shown in Figure 4(b) has a feature that the materials are left on the dried subdomain while the wet subdomain is shrinking. Therefore, this is *not* de-pinning, and different from what has been discussed in the existing reports. Figure 5 shows the time evolution of the flow velocity fields for samples A and B. Sample A shows the typical coffee-ring phenomena, where the flow is directed radially outwards, the speed is higher in the vicinity of the droplet periphery, and the speed increases at the final stage of the drying process, which is called the ‘rush-hour’ [14]. On the other hand, the time evolution of flow field for sample B starkly contrasts with that of sample A. The distribution of velocity fields changes with time, and the flow speed is higher in the inner part of the sample compared to the periphery. The speed up at the final stage of drying process is not observed, but the area that exhibits significant flow speed shrinks with time. This difference does not only indicate the presence/absence of the coffee-ring effect, but the qualitative difference of drying process as mentioned above and observed in Figure 4.

**Figure 7.**
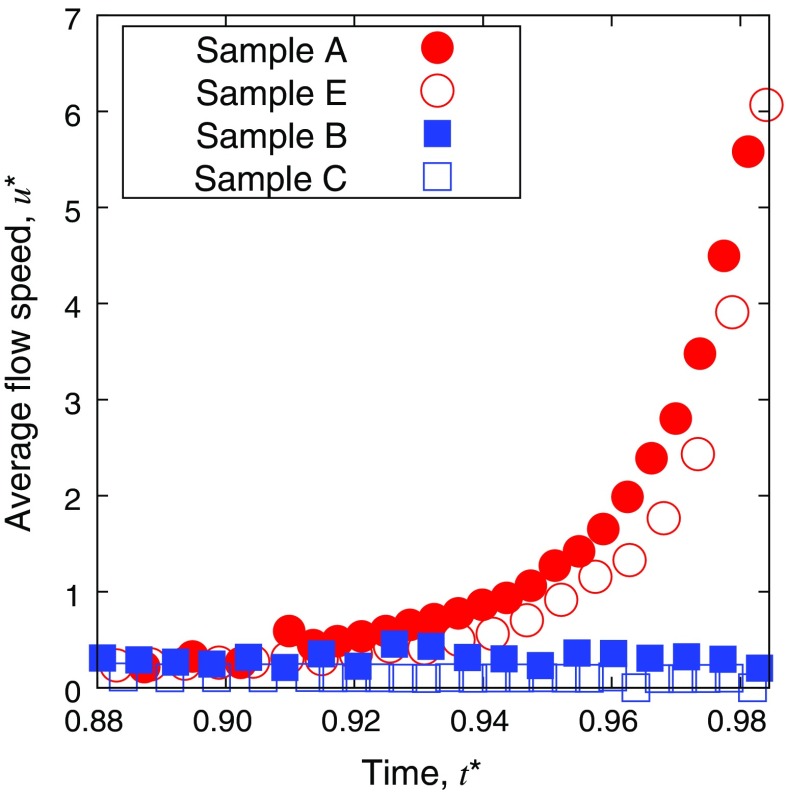
Time evolution of the flow speed for the samples with and without CNF. The averaging to extract 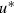 is based on the significant magnitudes of velocity data beyond the possible measurement error of PIV.

The velocity fields in the later stage of the drying process before its completion for different conditions of the samples are summarized in Figure 6. Comparison of color mapping reveals that the flow speed in samples B, C, and D was clearly much lower than in samples A and E. From a comparison of Figure 6(b) and (c), it can be observed that the higher concentration of CNF leads to even lower flow speed. This is further quantitatively confirmed from Figure 7, where instantaneous average of flow speeds in a droplet of each sample is shown as a function of time. Samples A and E show the clear emergence of coffee-ring phenomena with a rush-hour stage. Slight yet significant difference of speed can be confirmed between samples A and E. The increase of particle concentration slows down the flow, which is mainly attributed to the increase of effective viscosity by the concentration. On the other hand, samples B and C do not exhibit a flow acceleration at the last stage of drying. Their relatively uniform patterns indicate the absence of coffee-ring and rush-hour phenomena. It should be noted that the averaging to derive 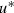 is based on the sufficient signal-to-noise ratio beyond the threshold value, which distinguishes the wet subdomain of Figure 4 from the dried subdomain, and accordingly the arrowed subdomain of Figures 5 and 6. Therefore, the substantially low 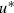 does not include the influence of dried subdomain. The higher concentration of CNF leads to lower speed of flow in the drying process, and in fact the flow speed of sample C is rather negligible compared to others. On the other hand, comparison between Figure 6(c) and (d) indicates that the 0.1 wt% (or less) of the CNF is sufficient to suppress the coffee-ring effect, at least in our system. The comparison of samples C and E in Figures 6 and 7 shows the remarkable difference of flow in the drying process in spite of the same total concentration of dispersed materials in a unit of wt% while the ingredient is different. Furthermore, it is worth noting that the concentration of CNF in sample B is only 1/10 of that in sample C and the total weight concentration of dispersed material is lower than E, but the rush hour is sufficiently suppressed.

## 4. Conclusions

The addition of a small amount of CNFs into an aqueous dispersion of colloidal particles suppresses the coffee-ring effect in the drying process. The CNF addition is much more effective than the increase of dispersed particle concentration, and the resulting colloidal film is more uniform compared to those prepared without the CNFs. These characteristics are useful as an environmentally friendly methodology for the suppression of coffee rings in applications ranging from printed electronic components to the fabrication of metamaterials. In addition, CNFs can also serve as the substrate in flexible electronic devices.

We studied the basic mechanism of the drying process by two types of computational data analysis, which were complementary and agreed with each other. The observation of time-differential images revealed the qualitative difference in the drying process. Without CNFs, a liquid droplet almost entirely covered the sample until the last stage of drying. Meanwhile, a particle dispersion with CNFs exhibited a shrinkage of liquid subdomains inside the sample. The drying dynamics was further examined by PIV. CNF-free particle dispersions exhibited a drastic acceleration (‘rush hour’) of the radial outward flow with elevated speed at the periphery at the final stage of drying. On the other hand, samples mixed with CNF did not show the rush-hour phenomenon, and the speed at the periphery was negligible at the final stage of drying. The concentration of CNFs inevitably increased at the final stage due to the water evaporation. The highly hydrophilic assembly of CNFs mechanically hindered the fast transport of water.
